# Exploring the public health potential of RED January, a social media campaign supporting physical activity in the community for mental health: a qualitative study

**DOI:** 10.1016/j.mhpa.2021.100429

**Published:** 2021-10

**Authors:** Catherine Wheatley, Margaret Glogowska, Afroditi Stathi, Claire Sexton, Heidi Johansen-Berg, Clare Mackay

**Affiliations:** a Wellcome Centre for Integrative Neuroimaging, Nuffield Department of Clinical Neurosciences, University of Oxford, John Radcliffe Hospital, Oxford OX3 9DU, UK; b Nuffield Department of Primary Care Health Sciences, University of Oxford, New Radcliffe House, Woodstock Rd, Oxford, OX2 6GG, UK; c School of Sport, Exercise and Rehabilitation Sciences, University of Birmingham, Birmingham, UK; d Wellcome Centre for Integrative Neuroimaging, Oxford Centre for Human Brain Activity, Department of Psychiatry, University of Oxford, Oxford, United Kingdom

**Keywords:** social prescribing, social media, mental health, physical activity, self-determination

## Abstract

**Statement of problem:**

RED January is an annual social media campaign challenging individuals to be physically active every day during January, and highlighting the potential for improvements in mood and wellbeing. Our aim was to explore elements of the challenge that motivate engagement with, and sustained participation in, physical activity for mental health.

**Method:**

RED January registrants (n= 55,772, female = 45,802; 82%) were invited to take part. Volunteers supplied information on sex, age band and moderate-to-vigorous physical activity in the past week. Forty registrants (24 female), recruited in December 2019 using a purposive sampling approach to identify a maximum-variation sample, participated in semi-structured interviews (31 face-to-face) after completing the challenge. The resulting transcripts were thematically analysed, using the Framework method.

**Results:**

Two main themes relating to motivation were identified. ‘Pleasure’ referred to how daily activity promoted physical enjoyment and positive affective states via engaging with the environment, finding mental space and peace, and enjoyable social interactions. ‘Purpose’ referred to the experiences of engaging with the campaign, and observed changes in health outcomes. These included setting flexible and appropriate goals, measuring and reviewing progress, noting wider biological and behavioural changes, and receiving support from the social media community. Points for consideration were feelings of failure when not achieving self-imposed targets, the unintended facilitation of obsessive exercising, and social media posts that triggered negative thoughts among a minority of participants.

**Conclusions:**

Findings suggest RED January may have potential as a public health resource. The challenge might not suit individuals with severe disorders.

## Introduction

Poor mental health is a major cause of disease burden worldwide (Vos et al., 2015), and in the UK one adult in six has a common mental disorder (McManus, Bebbington, Jenkins, & Brugha, 2016). Recent analyses comparing trends in mental health before and during the Covid-19 pandemic have revealed an overall deterioration (Pierce et al., 2020). Furthermore, ease of access to mental health care has declined in this period (Kola, 2020), indicating a growing need for affordable, accessible and effective support for population mental health.

Regular physical activity has been shown to have protective effects against depression (Rhodes, Janssen, Bredin, Warburton, & Bauman, 2017). It can also promote aspects of mental health - a positive construct comprising social, emotional and psychological wellbeing (Keyes, 2002) - by lowering anxiety and increasing self-esteem (Biddle, 2016; Rebar et al., 2015) and satisfying basic psychological needs for autonomy, relatedness and competence (Teixeira, Carraça, Markland, Silva, & Ryan, 2012). According to public heath guidelines, for good physical and mental health, adults should aim to be active every day, and to accumulate at least 150 minutes per week of moderate-intensity activity (such as brisk walking) or 75 minutes of vigorous activity (such as running), or a combination of these (World Health Organization, 2016; The Chief Medical Officers for England, Scotland, 2019). The relationship between physical activity and mental health may also be influenced by contextual factors including exercise type and enjoyment (Teychenne et al., 2020).

Despite numerous interventions promoting population-level physical activity, there is no clear evidence showing the most effective approach (Rhodes et al., 2017). Motivating people with low mood to exercise can be particularly challenging due to emotional and affective barriers including low self-efficacy, lack of enjoyment and feelings of fatigue (Glowacki, Duncan, Gainforth, & Faulkner, 2017; Vancampfort et al., 2015). Interventions in primary health care settings, such as brief advice or referral to community-based programmes, are cost-effective (Abu-Omar et al., 2017; Garrett et al., 2011), with some evidence for clinical effectiveness (Rhodes et al., 2017). Walking and running groups, and mass community events such as marathons or charity walks, which use social engagement to motivate, have been suggested as potential public health resources in this context (Bauman, Murphy, & Lane, 2009; Reece, Quirk, Wellington, Haake, & Wilson, 2019). Interventions underpinned by models of behaviour change have stronger effect sizes than a-theoretical approaches (Webb, Joseph, Yardley, & Michie, 2010), and can be evaluated and replicated more accurately (Abraham & Michie, 2008).

RED January (www.redtogether.co.uk; @redjanuaryuk) a social media campaign which challenges people to do something active ‘every day, your way’ for a month to ‘beat the blues away’ is a free, community-based resource with potential for social prescribing (signposting patients to non-clinical community activities). The campaign is unique because its explicit focus is encouraging daily physical activity to improve mental health and wellbeing, and because it encourages people to set their own exercise goals.

**Figure 1:**
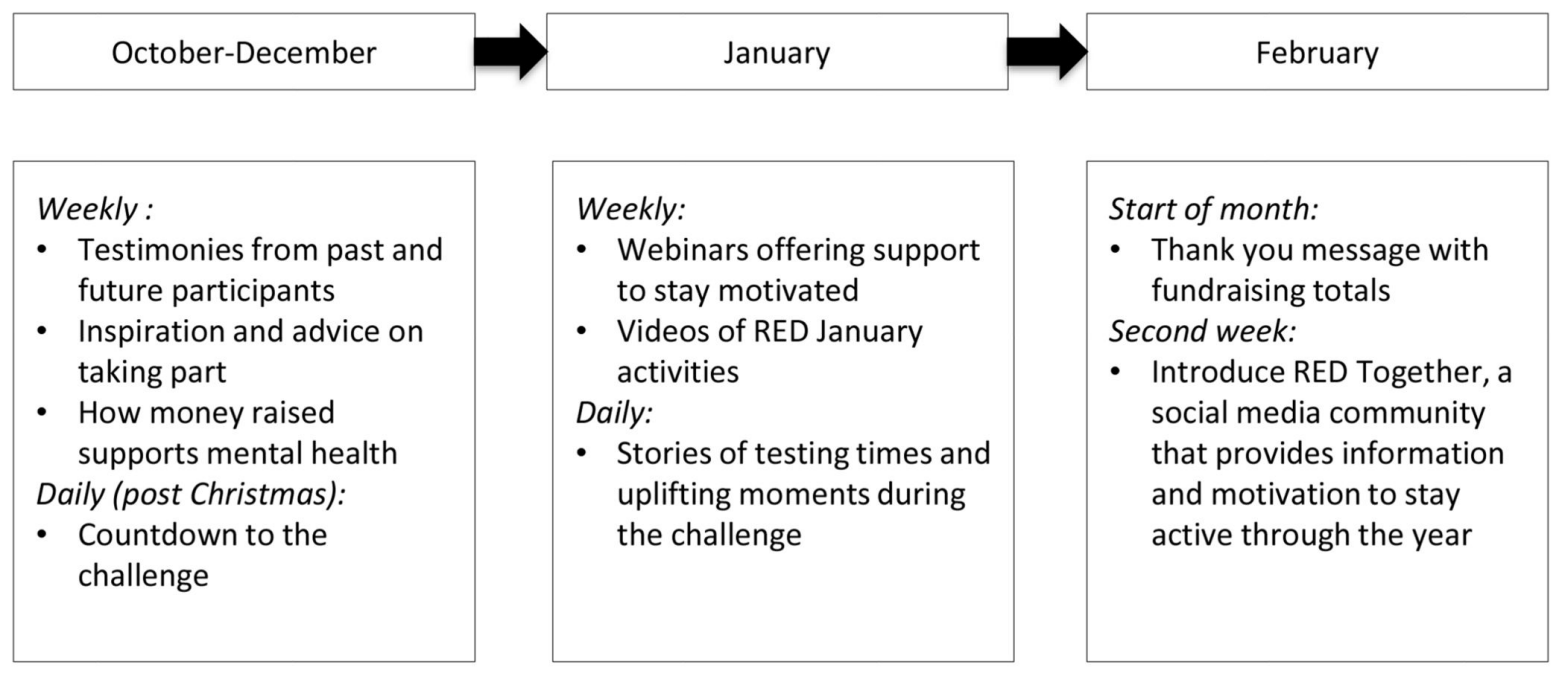
Timeline showing RED January campaign's social media content

The initiative, based in the UK but open to anyone, was launched in 2016 and has been promoted by the UK mental health charities Mind and Sport in Mind. It is organised around social marketing campaign principles rather than behaviour-change theory: there is a clear call to action promoted with strong messages and images shared online and via email. From the start of October when registration opens, registrants receive regular support emails and can follow social content (see Figure 1 for broad content and timelines). The organisation also moderates social media communities on Facebook, Twitter and Instagram. Some registrants opt to raise sponsorship money. Evidence from similar one-month health-behaviour challenges, such as Dry January, which encourages temporary alcohol abstinence, and Stoptober, which promotes smoking abstinence, is broadly positive (Brown et al., 2014; de Visser & Piper, 2020).

Whether and how RED January might have potential as a public health resource by promoting the adoption and maintenance of physical activity in the community for mental health has not been investigated. Qualitative methods are indicated for answering questions about personal experiences and perspectives, including the potential strengths and weaknesses of proposed interventions (Flick, 2018). We therefore employed a qualitative methodology to explore elements of the challenge that motivate engagement with physical activity for mental health, and those that contribute to sustained participation, using semi-structured individual interviews.

## Method

### Design

A qualitative interview study embedded in the RED January research project, reported in accordance with COREQ (COnsolidated criteria for REporting Qualitative research) guidance (Tong, Sainsbury, & Craig, 2007). The University XXX Research Ethics Committee granted approval to conduct semi-structured interviews with adult RED January registrants, either face-to-face or using videoconferencing [R67006/RE001]. All research was performed in accordance with the Declaration of Helsinki.

### Participants and interview schedule

In December 2019 all RED January registrants were invited, via an email from the organisers, to take part in the study. To inform purposive sampling, volunteers were asked to complete a very brief online survey of sex, age, and a single-item measure of physical activity that is suitable for screening: “In the past week, on how many days have you done a total of 30 minutes or more of physical activity, which was enough to raise your breathing rate?” (Milton, Bull, & Bauman, 2011). Eligible volunteers were at least 18 years old, living in the UK and had given informed consent to a recorded interview. Participants were told data would be anonymised and shared with researchers.

To ensure a variety of participants reflecting the range of registrants, we used a sampling matrix including sex, age band, previous participation in RED January and days per week of physical activity: volunteers were dichotomised as either active (≥5 days, ie 150 minutes, per week) or inactive (all others). We set a target of recruiting 20 females and 20 males, aiming to include individuals from each age group and activity category. The target sample size was pragmatic, reflecting an assumption that interviews should take place no more than a month after the end of the challenge, to ensure registrants’ recollections were fresh. Selected volunteers received an email and a follow-up telephone call: individuals who declined or did not respond were replaced by the next volunteer matching the appropriate category. All others received an email thanking them for volunteering.

Immediately before interview, we took brief, in-person measures of participants’ mental health and wellbeing to guide the interpretation of qualitative data. (Nine participants interviewed via videoconference completed the measures online). Depressive symptoms were measured with the Patient Health Questionnaire-9 screening tool (Kroenke & Spitzer, 2002) (PHQ-9; sum of 9 items, range 0-27; scores of 5-14 indicate mild-moderate depression and 15-27 severe depression). The PHQ-9 has been shown to be a reliable and valid tool for identifying and grading depressive symptoms (Kroenke, Spitzer, & Williams, 2001). Subjective wellbeing was assessed with 4 items from the Office for National Statistics (ONS) Annual Population Survey National Wellbeing Measures (Office for National Statistics), which are widely-used, and recommended for public policy research (Dolan & Metcalfe, 2011): ‘Overall, how anxious did you feel yesterday?’ (range 0-10; scores 0-1 low); ‘Overall, how satisfied are you with your life nowadays?’; Overall, to what extent do you feel that the things you do in your life are worthwhile?’; Overall, how happy did you feel yesterday?’ (range 0-10; scores 9-10 high).

A female postdoctoral researcher trained in qualitative approaches and not known to participants conducted interviews using a semi-structured schedule of questions and probes asking about the experience of taking part in RED January; about mood and wellbeing during the month; and about outcomes of the challenge including future activity plans (schedule available in the Supplementary File). The topic guide underwent minor revisions during the interview process to probe emerging topics including recording activity and wider health outcomes. All participants were provided with contact details for the Samaritans’ mental health helpline, a condition of ethical approval. Interviews were digitally recorded and transcribed verbatim, with personal information deleted to preserve anonymity.

### Analysis

An experienced qualitative researcher employed thematic analysis (Braun & Clarke, 2006) to explore patterns of meaning in the data, using the Framework method (Gale, Heath, Cameron, Rashid, & Redwood, 2013; Ritchie, Spencer, Bryman, & Burgess, 1994), a flexible but structured approach to data management and analysis. The process began with reading and loosely coding transcripts while interviews were still taking place, and noting recurring points and key observations. These notes were used to develop a thematic framework, into which participants’ data could start to be organised under provisional topic headings during the interview stage. Our framework was organised around contextual questions – such as what are participants’ experiences and goals during the challenge – and evaluative questions, such as why and how does the challenge ‘work’? Data were then abstracted from each transcript and organised into a series of charts representing these topic headings and sub-headings. Charts were refined and headings were merged and rearranged to capture major categories (eg ‘wellbeing and exercise’) and sub-categories (eg ‘paying attention to the moment’). Finally, in the interpretation phase, charts were reviewed to examine patterns in the data and connections between ideas, and to identify final themes. A second, senior researcher rigorously challenged, discussed and agreed the interpretation of results with the primary analyst. NVivo 12 was used to organise and manage data during coding, and Excel 2011 was used for charting. For practical reasons, participants were not invited to provide feedback on the findings. Data is presented using pseudonyms.

## Results

Of the registrants (n= 55,772; female = 45,802 or 82%; age 25-64 = 49,831 or 90.2%), 474 volunteered for interview (0.9%). Of these, 112 were invited to the study: 48 did not respond, 16 declined and eight withdrew (reasons not recorded). Forty individuals were interviewed (24 female; 31 face-to-face) between 3 February and 2 March 2020: interview length ranged from 15 to 42 minutes. Interviews revealed that four participants had withdrawn from the challenge during January: of these, two screened for severe depression. Table 1 shows participants’ characteristics.

We identified two main themes describing experiences that appeared to motivate engagement with, and sustained participation in, physical activity for mental health during RED January: ‘pleasure’ and ‘purpose’. These ideas, incorporating several sub-themes, have areas of overlap and are described in Table 2. For example, statements about feelings of calmness or euphoria while exercising were often linked to statements about mental health goals and outcomes. We report these themes separately, while acknowledging their links.

### Theme 1 Pleasure

This theme describes physical sensations, affective states and emotions relating to exercise during RED January, and how these motivate exercise habits. The challenge brought participants’ attention to their physical activity and associated mental states. These feelings were familiar and welcome for some, but others were exploring links between exercise and mental health for the first time.

#### Sub-theme

*Focus on physical sensations*. When talking about exercise, many participants – especially females - referred to physiological feelings of regular movement and the rhythm of their heart, breathing and limbs, and of movement through air or water. A few described the pain of exercising through injury or to the limit of their endurance. The words ‘endorphins’, ‘buzz’ and ‘euphoric’ came up repeatedly. Descriptions of feeling ‘calm’, ‘quiet’ and ‘focused’ were also common. Paying attention to these feelings, and their link with positive mood states, was important. For some, focusing on physical sensations was a form of meditation:

*“I have no thought and no awareness of anything else but just being in the pool and just swimming…”* (Patricia, 35-44, inactive, mild-moderate depression)

A few experienced these sensations as relief from negative mental states. For example, Neil (35-44, active, mild-moderate depression), described overcoming feelings of dissociation: “I can feel myself within my body… when I’m using my weights.”

#### Sub-theme

*Engaging with environment*. Many took pleasure in fresh air, nature and the environment while exercising. Jane (25-34, active, mild depression) described “a couple of really lovely mornings when it was like frosty, but the sky was just lovely, a beautiful sunrise and stuff. “ Another reported while cycling:

*“…you see foxes and hares and you know on a nice frosty morning it would be absolutely brilliant*.” (Alan, 65+, active, minimal depression)

The urban outdoors was also pleasurable, said Portia (35-44, inactive, minimal depression): “Instead of taking the tube…it’s much nicer walking to work and being outside.” Some mentioned making an effort to be ‘conscious’ or ‘mindful’ of their environment. Bea, (45-54, inactive, mild-moderate depression) said: “I try and actually be a bit more present in the moment. “ Participants seemed aware that exercising in pleasant surroundings was linked to positive mood:

“*It makes you feel better just being outside in the fresh air and looking at trees or birds and gardens and stuff,*” (Ursula, 45-54, inactive, mild depression)

##### Sub-theme

*Mental space, clarity and peace*. Exercising during RED January offered space and freedom to escape from worries or responsibilities. Tracey, (45-54, inactive, minimal depression) described exercising as “a cut-off, it gives you a clean mind and refreshes you.” Orla, (35-44, inactive, mild-moderate depression) said: “…after I’ve gone for a jog and a walk I feel so, like I feel fresh, I feel happy.” Space was a common reference:

“*I use it as my headspace…if I’ve had a tough day because of my job…I can stop anywhere and just get out for a run*.” (Graham, 45-54, active, minimal depression)

Exercising offered time for reflection, resolving problems or clearing the mind of negative feelings. “You kind of get a chance to think about it all and rationalise it and sort of sort it out,” said Bob (35-44, active, minimal depression). For others, exercising brought peace. Harry, (25-34, inactive, minimal depression) said: “it [running] is a bit of a relief from just every day noise in the head”. Others described feeling calm:

*“My mind was quiet for most of the month…There’s a switch that goes and during the exercise … all the doubts and all the voices… they get quiet*.” (Carla, 35-44, active, minimal depression)

For a few participants, time alone exercising could be a negative experience. “Being in my own head with no distractions is quite tough for me,” said Neil, (35-44, active, mild-moderate depression).

##### Sub-theme

*Social interactions*. Supporting others to get more active gave pleasure to many (see A Social Media Challenge sub-theme). Lee, (25-34, inactive, mild-moderate depression) persuaded a flatmate to participate: “The fact that he was out being active and doing it made me feel great.” Another described running with a housemate who had been feeling low:

*“I was doing that hill and I was like* oh, he’s going to hate me for this *and he came through it like absolutely beaming…I feel actually really good for that.”* (Freddie, 35-44, active, minimal depression).

Social aspects of exercising were pleasurable, especially for female participants. Rose, (55-64, active, minimal depression) described meeting friends after swimming. “…sometimes there’s a bit of a social side to it. So, that’s really nice and I’ve enjoyed that.” Some enjoyed giving and receiving support via RED January’s social media platforms. Alison, (45-54, active, minimal depression), said: “The more people can feel happy about themselves and others the more that happiness can kind of spread.” Another simply said:

“*It’s nice to encourage peopl*e,” (Bob, 35-44, active, minimal depression)

Some described organising RED-themed events in the community or the workplace. Sarah, (25-34, active, mild-moderate depression), said: “We had a little group of us that sort of talked internally about RED January and exercise so that was really good.” A few admitted anxiety and sometimes distress after taking a lead. Alan, (65+, active, minimal depression) said: “it’s…not how…positive I feel before, it’s how disappointed I am if it doesn’t come off.”

### Theme 2 Purpose

This theme describes how RED January motivated participants to get and stay active. Although the challenge is flexible, most committed to specific exercise objectives. Many aimed to establish new, long-term exercise habits, for physical health, rehabilitation or mental wellbeing, often in the context of a busy lifestyle. Some wanted to help others to get more active by organising work or community events. Behaviours the challenge encourages – recording activity and joining RED January social media channels - motivated many to work towards these goals. Several participants with depressive symptoms described a history of struggling to find motivation to exercise, despite understanding the potential benefits.

#### Sub-theme

*Flexible goals that fit*. Participants’ interpretations of ‘every day, your way’ produced goals tailored to individual circumstances and interests. Targets involving running, walking, or both, were common. Many also included one or more of swimming, cycling, yoga or fitness activities, while a few mentioned indoor rowing or golf.

There was awareness of the injury potential of being active daily, especially from a low start. Several participants described goals involving organising and leading exercise for mental health at work or in the community. Most participants were flexible about type or duration of activity. Katie, (45-54, inactive, minimal depression) ran every day, but was flexible about duration. “…maybe a short run around the block. If I had more time it would be a 5K run...”. Another described variable intensity:

*“I wanted to be active every day, I knew that probably some days my activity would be more around like something like a brisk walk…gyming during the week and then sort of just making sure I’m moving at the weekend*.” (Kelvin, 25-34, inactive, mild-moderate depression)

Most embraced the flexibility, noting it encouraged daily activity:

“*Little and often, every day…I didn’t want to set myself up to fail,”* (Leanne, 45-54, inactive, mild-moderate depression).

Others felt flexibility helped them adapt to exercise barriers - typically work or family commitments, injury, fatigue or low motivation. As Dora, (25-34, inactive, minimal depression) surmised, “[For] people who do long shifts or have other commitments it’s quite a lot of pressure to exercise every day. But by saying *something* it means that, you know, ten minutes of yoga on YouTube - you’ve still done more [than otherwise].” Freddie, 35-44, active, minimal depression), an endurance exerciser, said: “[I] deliberately didn’t push it because I wanted to last the whole 31 days.”

A few struggled with self-directed goals, including Gina, (35-44, inactive, minimal depression) who contacted RED to confirm that 20 minutes a day was acceptable. Zeena, (18-24, inactive, minimal depression) felt that counting routine physical activity, including cycling to work, was not sufficiently stringent: she pulled out of the challenge as a consequence. Most were happy to forgive themselves a missed or light-active day:

*“You know, don’t worry about it. Just doing something different to what you did yesterday takes you in a different direction, makes you feel better”*. (Graham, 45-54, active, minimal depression)

A minority expressed feelings of failure from having committed to something too taxing. “I’m disappointed with myself,” said Euan (55-64, inactive, minimal depression), one of two participants who did not complete the challenge due to travel and family commitments. Emma (35-44, inactive, severe depression), who also withdrew, described being too depressed to leave the house for exercise or any other reason: “As time went on…I was beating myself up about not doing [the challenge].” Maria, (25-34, inactive, mild-moderate depression) described a low point on realising she could not run every day, although she found other ways to be active. “That was...setting too high expectations on myself. And negative talk to myself about not being able to see it through.”

Committing to RED January, despite its flexibility, seemed to bolster willpower and self-accountability. As Nicola (45-54, active, minimal depression) said: “it took the decision-making out of what exercise I was going to do, *oh, can I be bothered*? Another described her strengthened resolve:

“*There have been days when I’ve got home and thought* oh, I can’t be bothered… today has been awful and I’ve earned the right to sit on the sofa. *But then that other voice has told me* no, just go out, 10 minutes that’s all you need.” (Maria, 25-34, inactive, mild-moderate depression)

#### Sub-theme

*Multiple aims and outcomes*. RED January aims to get people active ‘to beat the blues away’, but participants also had long-term self-care aims. Many wanted to take responsibility for their physical and mental health:

*“I think there comes a choice where it’s a decision. And you just say ‘*enough…I don’t want to be like this,’*“* (Vanessa, 45-54, inactive, mild-moderate depression)

Performance aims included ‘improved times’ and ‘build up my distance’ while physical health aims involved ‘losing weight’ and ‘get fitter’. Sometimes, these followed concerns about disease and ageing. Willa (65+, inactive, mild-moderate depression) wanted to lose weight after being diagnosed as pre-diabetic. Many also aspired to long-term behaviour change. Zeena (18-24, inactive, minimal depression) said: “I’ve started a…potential forever job…if I don’t get into good habits at the start then I don’t want to wake up in 10 years, 20 years and feel regret.”

Some experienced exercisers described the challenge as a gentle way back into regular activity after injury. “I thought *I’m going to use this as my kick start back into movement*,” said Felicity (35-44, inactive, mild-moderate depression), a former runner who took up yoga and walking. Others used the challenge to explore new ways to tackle barriers to exercise:

“*Work gets in the way [*of exercise*], family, kids and everything…it is vitally important to…look after yourself both physically and mentally*.” (David, 35-44, inactive, minimal depression).

Stabilising or improving mood and wellbeing was a common aim. Several wanted to explore and understand how physical activity was related to their personal mental health (see Keeping a Record Motivates). Kelvin (25-34, inactive, mild-moderate depression) wanted to improve physical self-esteem and reduce anxiety. “[The challenge helps to]…kick start…a change in how you view yourself. Or how you view your mental health and how you want to look after yourself.” Another noted:

“*RED January was for me a goal and a stepping stone…to psychologically build myself back up that I’ve given myself the confidence that I can do this [*exercise*],”* (Ian, 45-54, inactive, mild-moderate depression).

Many described using the challenge to tackle seasonal low mood:

“*January 1st it’s…daunting. You’ve got…social pressure to be setting resolutions, doing better, bigger, faster, stronger…having a focus such as maintaining a level of exercise is always good*,” (Alison, 45-54, active, minimal depression).

A few considered their unhealthy relationships with exercise. “In January I probably scaled back a little bit and I was quite mindful that I was excusing an obsession rather than [exercising] for enjoyment,” said Sarah (25-34 active, mild-moderate depression), who admitted running despite a stress fracture in the previous year and who described fighting the urge to engage in multiple daily exercise sessions. Another, Gina, (35-44, inactive, minimal depression) a former marathon runner with multiple injuries, described her goal as *not* running every day – substituting short Pilates or yoga sessions - to avoid additional strains: “[At the start of February] I had to force myself to have the day off,” she said.

#### Sub-theme

*Measuring/reviewing progress*. Most participants recorded their activity during the month, and some noted their mood. Many used a Fitbit, Garmin or other device to track activity. A few crossed off each day they exercised on the RED January calendar. Of these, some shared their activities on Strava or RED’s social media channels. Participants found keeping a record was motivational. For some it denoted task completion:

“*I like to be able to cross that checklist off and go right, that’s done*,” (Lee, 25-34, inactive, mild-moderate depression).

For Alison (45-54, active, minimal depression), “filling in the diary…helped with my self-discipline.” Tangible proof of achievement was important and uplifting for many. Euan (55-64, inactive, minimal depression), who described filling in a calendar during the 2019 challenge, acknowledged that “the challenge was really in my own head. But I could prove it if I wanted.” Another sewed a button on her T-shirt every day she was active:

“*[I] felt really proud and that I’d got something to show for it,”* (Patricia, 55-64, inactive, mild-moderate depression)

Many had time or distance-related goals, so reviewing progress was important. Ian (45-54, inactive, mild-moderate depression), said: “There’s an expectation of looking forward to seeing how many lengths I swam.” Many reported being generally more conscious of exercise, mood and how they were related (see sub-theme: Promotes wider health outcomes). A few made diaries of health behaviours during the month:

“*It was a scientific test of like how much it was benefitting me*.” (Carla, 35-44, active, minimal depression)

Sharing progress kept some accountable. Dora, (25-34, inactive, minimal depression) said that after posting her activity daily on Instagram “someone is going to notice if I don’t post day 11.” Accountability was also important for the handful raising sponsorship money.

Some said keeping a record fostered routine, which was important for a few participants with poor mental health. Jane (25-34, active, mild depression) said the tick-list approach to exercise was important for her recovery from depression: “I knew that like each day I would have to tick those boxes off.” Another said:

“*My mental health is always better if I’m in a routine - and building going to the gym into my routine in the past six weeks has meant I’ve kept a really good track.”* (Kelvin, 25-34, inactive, mild-moderate depression).

Some felt diaries over-emphasised goals - and potential failure. “I don’t want to get too obsessed with how many steps I’ve walked each day….if you’re quite an anxious person I don’t think that’s really helpful,” said Bea (45-54, inactive, mild-moderate depression). Katie (45-54, inactive, minimal depression) did not monitor exercise because she would “get frustrated if I hadn’t hit targets I’d met the week before. And…I’d be more inclined to give up, so, I didn’t …the main goal was to do something every day.”

#### Sub theme

*Promotes wider health outcomes*. Habitual physical activity was linked to positive change in other health outcomes, notably fitness, sleep and diet, according to many, which motivated participants to maintain their exercise habits. Some mentioned biological mechanisms. Several reported that increased activity led to better sleep quality, and that feeling less tired improved their mood:

*“…doing exercise every day - it did tire me out - so then when I went to bed I’d just fall asleep… all of these things kind of add up and contribute to a generally better mood*.” (Harry, 25-34, inactive, minimal depression).

A few said it helped with symptoms of menopause, including low mood. Others noted weight loss and changing body shape, which was linked to higher self-esteem. Others described behavioural mechanisms leading to a positive health changes. A number of REDers had also taken part in Dry January and/or Veganuary. Several swapped alcohol for exercise:

“*It* [alcohol consumption] *was on my mind when I signed up for RED January. If I commit to do this I won’t be able to just get drunk as much as I was doing*.” (Leanne, 45-54, inactive, mild-moderate depression)

Harry (25-34, inactive, minimal depression) described how cycling to and from the office led to him skip after-work drinks. “Exercise helps steer other parts of my life into a better place which in turn helps mental health…my mood definitely improved during January.” A few noted benefits from swapping screen time for exercise. “Having that 20-minute walk after work and not on my phone or checking emails…I would feel It’s not as bad as it felt when you first left the office,” said Maria (25-34, inactive, mild-moderate depression). A few described how committing to daily activity can lead to negative behaviour-change. “I will sacrifice the sleep for exercise…But then I get to a point where I sort of get physically exhausted and then it sort of spirals a bit out of control,” said Sarah (25-34, active, mild-moderate depression).

#### Sub-theme

*Social media support*. RED January’s social media community was important for many, although a substantial minority chose not to join. Users found the tone of communications ‘honest’, ‘positive’, ‘safe’ and ‘supportive.’ Of those who engaged, many found it encouraged them to exercise:

“*If someone has been out first thing in the pouring rain, they’ll put photos and stuff [on Facebook], it’s quite motivational. I think* right, well it’s sunny now I’ve got no excuse,” (Nicola, 45-54, active, minimal depression).

Others found support during low periods. “There are other people out there experiencing it as well as you...you’re not alone,” said Alison, (45-54, active, minimal depression). Dora (25-34, inactive, minimal depression), stressed that promoting exercise for mental health can be difficult for those with severe problems. “For them it belittles those who need medication, it makes them lack self-worth.” A few, who screened with depressive symptoms and described mental health problems, found the emphasis on others’ struggles difficult:

“*Sometimes it can get a little bit heavy, triggering if you like*,” (Vanessa, 45-54, inactive, mild-moderate depression).

For others, the challenge was private and individual. Harry (25-34, inactive, minimal depression), said “For me it’s more just a personal thing so I didn’t really bother.” Christopher (55-64, inactive, mild-moderate depression) was typical of some older participants: “Sharing it with the general public - no real interest.” The social capital acquired from helping others was important for some. Participants who had used exercise to deal with their own mental health problems described the importance of sharing their experience:

“*It’s quite nice to give some knowledge and advice...I shared about* [the death of her son] *…and it encourages other people to share it and then people actually realise they’re not isolated,”* (Rose, 55-64, active, minimal depression).

## Discussion

Participants described how elements of the RED January challenge grouped under ‘pleasure’ and ‘purpose’ motivated engagement and sustained participation in physical activity in the community for mental health. Interviews revealed that exercise self-efficacy, challenge goals, physical activity volumes and mental health and wellbeing varied widely among participants. These included experienced runners, gym members, and individuals returning to exercise or starting regular physical activity. Half reported at least mild depression at the time of interview, while many reported relatively low levels of wellbeing, and a small number described exercise-related anxiety or obsessive thoughts during interview. Neither of the two participants who scored for severe depression completed the challenge. Notably, RED January appears to use social media channels to create a supportive online community, suggesting the challenge could support physical activity when social distancing is required.

RED January is a social media campaign based on marketing principles rather than theoretical models of health behaviour change. Nevertheless, sub-themes we identified aligned with components of Self-Determination Theory (SDT) (Ryan & Deci, 2017) and Social Cognitive Theory (SCT) (Bandura, 1986), two overlapping and mutually compatible theoretical perspectives that are often used in exercise interventions (Rhodes, McEwan, & Rebar, 2019) and whose components are targeted in public health interventions (Windle et al., 2008). This finding is important because interventions that target theoretical constructs - whether deliberately or not - have stronger effect sizes than a-theoretical approaches (Webb et al., 2010), and can be evaluated and replicated more accurately (Abraham & Michie, 2008). Detailed explanations of these models are available elsewhere (Rhodes et al., 2019): we restrict our discussion to key components the challenge appears to target.

SDT identifies three basic psychological needs – autonomy, competence and relatedness - the satisfaction of which promotes wellbeing outcomes and improves the quality of behavioural motivation (Ryan & Deci, 2000). Two sub-themes, ‘flexible goals that fit’ and ‘multiple aims and outcomes’ described how the challenge satisfies the need for autonomy, which refers to feelings of volition and self-governing one’s behaviour and choices (Ryan & Deci, 2000). Interviewees noted how the freedom to adapt exercise goals to suit individual circumstances including time constraints, injuries or fitness levels allowed them to maintain daily activity. They also described a range of desired health outcomes that were self-selected and personally-relevant. In this respect, RED January contrasts with more prescriptive exercise interventions that specify training plans or exercise volume, such as the NHS “Couch to 5k” running app or *parkrun* (Reece et al., 2019).

Two further sub-themes, ‘measuring and reviewing progress’ and ‘promotes wider health outcomes’ described how the challenge develops feelings of competence (or mastery in SCT), which refers to learning new skills, or improving proficiency (Bandura, 1998; Ryan & Deci, 2000). Participants described how keeping a record of their exercise delivered tangible proof of progress and achievement in relation to health goals, and that online affirmation, part of the ‘social interactions’ sub-theme, could enhance associated feelings of capability. Some also reported that positive biological and behavioural changes as a consequence of being active every day provided evidence of a new or improving ability to manage their own health.

The ‘social media support’ sub-theme indicated that the challenge promotes relatedness, which refers to feeling a sense of belonging or connection to others (Ryan & Deci, 2000). These virtual relationships, which provided support for mental health problems and physical activity goals, appeared to be an important source of wellbeing for some. Evidence from this sub-theme also suggests the challenge can improve exercise self-efficacy, which is promoted by observing positive role-models, according to SCT (Bandura, 1986). Some described how reading social media posts from people who overcame barriers to exercise such as weather or low mood motivated them to maintain their daily routine. The ‘social interactions’ theme included evidence that some participants took pleasure in supporting others to get active, which appears to be a unique strength of the challenge.

Feeling states are largely absent from behaviour-change theories, but affect can be an important determinant of behaviour among people with depression (Glowacki et al., 2017): there is evidence that exercise enjoyment may positively influence the relationship between physical activity and mental health (Teychenne et al., 2020). The ‘pleasure’ theme describes how participants derived positive affect from exercise, further enhanced for some by a free choice of physical activity, such as exercising outdoors in natural surroundings, or with other people, or at key times such as after a stressful day at work. The ‘focus on physical sensations’ sub-theme described how most participants paid attention to positive physiological feelings and associated mood states during and after exercise, while the ‘engaging with the environment’ and ‘mental space, clarity and peace’ sub-themes described how physical activity improved wellbeing through mindfulness of the environment or quietening stressful or anxious thoughts. It seemed that active participants with minimal depression were particularly likely to take pleasure from social interactions.

Sub-themes also mapped onto the ‘Five Ways to Wellbeing’, a set of evidence-based public health messages endorsed by the NHS (New Economics Foundation, 2008). The advice – be active, take notice, give, learn and connect – is recognised as having potential to enhance population wellbeing. As well as the clear message to get more active, RED January also facilitates other ‘Five Ways’ behaviours. As described above, participants mentioned taking notice of, and pleasure in, feelings, moods, nature and the environment. Some found that the campaign encouraged them to give to others: participants derived social capital and fulfilment from encouraging inactive friends to get fitter or supporting others with mental health struggles. Interviewees with injuries, or who were returning to exercise, mentioned satisfaction from exploring new activities. Although connecting implies in-person interactions, RED January’s social media community appeared to be an important source of wellbeing for some.

### Points for consideration

Women account for a large proportion of RED January registrants. Female sex is associated with insufficient activity and poor mental health (Rhodes et al., 2017; Silva, Loureiro, & Cardoso, 2016): programmes that support women to get and stay active for mental wellbeing are therefore welcome. The campaign’s messaging emphasises the positive feelings and sense of community that taking part can deliver: we found evidence that women in particular appear motivated by pleasure derived from the physiological sensations of physical activity, and the social aspects of exercise.

Some participants reported negative experiences. For a small minority, the call to exercise ‘every day, your way’ represented either a strict, self-imposed target, or an ill-defined and insufficiently-challenging goal. One of the youngest participants found the absence of specific goals off-putting enough to withdraw, while others expressed feelings of failure after missing their targets. A few undertook strict exercise regimes that appeared to encourage anxiety or obsessive thoughts. Two participants with severe depression withdrew, one of whom described how failing to meet her exercise goals lowered her mood. Two reported that that engaging with the social media community about mental health struggles triggered distressing thoughts. These findings suggest the challenge might not suit individuals who are uncomfortable with self-determination or whose motivation to achieve exercise goals is regulated by self-control or internal rewards and punishments (Ryan & Deci, 2000). It is unlikely to be indicated for individuals with obsessive behaviours or severe anxiety or depression.

### Strengths and limitations

Interviewing a large and diverse sample shortly after the challenge ended, including four who did not finish and two with severe depression, allowed us to develop a comprehensive understanding of people’s experiences of RED January. Discussions with a senior researcher ensured a rigorous and trustworthy data analysis. Our final sample included one under-24 and two over-65s, reflecting the age profile of RED January registrants. But there may be more to learn about whether and how the challenge ‘works’ in these age groups. Also, our pragmatic sampling strategy might have excluded individuals whose mental ill-health prevented them from volunteering to take part. This might have restricted the range of experiences described: it is possible our sample was biased in favour of positive experiences. We opted not to screen for depression or anxiety at the point of volunteering to reduce participant burden, so we were unable to select specifically for individuals with low mood. We did not explore the extent to which participants continued to engage with RED January beyond February.

### Implications for interventions promoting physical activity for mental health

Our study identifies features that could be included in other community-based interventions. Autonomy to select personally-relevant exercise goals and adapt them to suit individual circumstances was important for participants’ motivation, as was boosting feelings of competence by recording and sharing progress towards those goals. RED January’s social media community fostered motivation by promoting relatedness: virtual relationships provided support for low mood and physical activity goals, although potential for negative experiences should be considered.

## Conclusions

This study highlights the potential of a one-month social media campaign that does not require face-to-face supervision to motivate people with low mood and some exercise self-efficacy to engage in regular exercise for mental health. In line with public health interventions using exercise to promote mental wellbeing, it can target common theoretical constructs. It might not suit individuals with chronic disorders including severe depression, anxiety and obsessive behaviours. Future research should focus on measuring the impact of participation on physical activity, mood and wellbeing during January and the extent to which it leads to long-term behaviour change.

## Supplementary Material

Supplementary: Semi-Structured Interview Schedule

## Figures and Tables

**Table 1:** Participants' age, activity level and mental heath measures by sex (n=40)

Characteristics	Male		Female		Total	
	n	%	n	%	n	%
Total	16	40	24	60	40	100
Age						
18-24	0	0	1	2.5	1	2.5
25-34	3	7.5	4	10	7	17.5
35-44	4	10	6	15	10	25
45-54	6	15	8	20	14	35
55-64	2	5	4	10	6	15
65+	1	2.5	1	2.5	2	5
**Activity Level in December**						
Active (≥ 150 mins/week)	5	12.5	7	17.5	12	30
Inactive (< 150 mins/week)	11	27.5	17	42.5	28	70
**Mental Health & Wellbeing at Interview**						
^1^Severe depression	1	2.5	1	2.5	2	5
^2^Mild-moderate depression	7	17.5	12	30	19	47.5
^3^Mimimal depression	8	20	11	27.5	19	47.5
^4^Low anxiety score	0	0	2	5	2	5
^5^High happiness score	6	15	2	5	8	20
^5^High life satisfaction score	0	0	4	10	4	10
^5^High life worthwhile score	5	12.5	2	5	7	17.5

1 PHQ-9 Score 15-27

2 PHQ-9 Score 5-14

3 PHQ-9 Score 0-4

4 ONS Wellbeing Item Score 0-1

5 ONS Wellbeing Item Score 9-10

**Table 2:** Themes and sub themes relating to participation in RED January.

Theme	Sub-theme	Points
1. Pleasure	Focus on physical sensations	Paying attention to physiological sensations Noticing links between sensations and mood
	Engaging with the environment	Noticing fresh air and nature Existing in the moment Noticing link between environment and positive mood
	Mental space, clarity and peace	Escape from worries or responsibilities Time to think constructively Quietens inner monologue
	Social interactions	Taking pleasure in supporting others to get active Enjoying others' company Online affirmation
2. Purpose	Flexible goals that fit	Frequency, duration and type of goals vary Goals can be adapted to suit lifestyle and injury Any exercise is better than none Feelings of failure when goals are too tough Committing to goals encourages self-accountability
	Multiple aims and outcomes	Losing weight, getting fitter, tackling signs of ageing Improving long-term exercise habits Stabilising and improving long term mood Tackling seasonal low mood
	Measuring/reviewing progress	Satisfaction - crossing exercise off a checklist Tangible proof of achievement Reviewing progress - and monitoring the benefits Fostering routine Can overemphasise goals and highlight failure
	Promotes wider health outcomes	Biological: better sleep quality and body composition Behavioural: focus on diet, alcohol and technology usage Can encourage obsessive exercise
	Social media support	Positive and safe social media community Offers motivation to exercise and support for low mood Engaging can boost social capital Social media can trigger negative thoughts or feelings
