# Supplementary material for: Exploring the public health potential of RED January, a social media campaign supporting physical activity in the community for mental health: a qualitative study
Source: Ment Health Phys Act. Author manuscript; Available in PMC 2022 Feb 10. (PMC7612346; doi:10.1016/j.mhpa.2021.100429)
Supplement: Supplementary: Semi-Structured Interview Schedule [file EMS141011-supplement-Supplementary__Semi_Structured_Interview_Schedule.pdf]

Semi-structured interview schedule

* I am recording the interview. We can stop at any time. If you find any questions difficult or upsetting, and you would like to speak to someone who can help, we can give you the number of the Samaritans helpline (116123).

**Topic**		**Indicative Questions**
Evaluation of RED January		Were you involved in any activities prior to the RED January? If yes, what were those activities?<br>What activity did you aim to do during January?<br>What activity did you actually do during January? (Type/frequency/intensity/duration)<br>- Probe: was it important to 'do s*omething* active every day, *your way*'?<br>Did you raise money, buy a T-shirt/merchandise, or receive a medal?<br>- Probe: To what extent was this important to you?<br>Were you active alone, or with family or friends, or a club or group, or the RED January online community?<br>How many days off did you have?<br>Tell me about your experiences during RED January:<br>- What were the best moments?<br>- What, if any, were the challenges of RED January?<br>- Did you face any barriers in your effort to increase your physical activity? If yes, what were those barriers?<br>Would you say that the experience of participation matched your expectations? Why do you think that was the case?
Mood and mental wellbeing		RED January is about getting active every day to *beat the blues away.*<br>Can you say more about what feelings you experienced during the challenge?<br>- Probe: Before, during and after activity
Outcomes	Engagement / Motivation	How do you feel about physical activity now you have finished the challenge?
	Self-esteem	How do you feel about yourself/ how do you value yourself now you have finished the challenge?
The future		What are your plans for activity in the future?<br>Will you take part next year?<br>Would you recommend RED January to friends?<br>Suppose that you were in charge and could make one change that would make RED January better. What would you do?
